# Human cytomegalovirus multiple-strain infections and viral population diversity in haematopoietic stem cell transplant recipients analysed by high-throughput sequencing

**DOI:** 10.1007/s00430-021-00722-5

**Published:** 2021-10-06

**Authors:** A. Dhingra, J. Götting, P. R. Varanasi, L. Steinbrueck, S. Camiolo, J. Zischke, A. Heim, T. F. Schulz, E. M. Weissinger, P. C. Kay-Fedorov, A. J. Davison, N. M. Suárez, T. Ganzenmueller

**Affiliations:** 1 Hannover Medical School, Institute of Virology, Hannover, Germany; 2 German Center for Infection Research (DZIF), Site Hannover-Braunschweig, Hannover, Germany; 3 Department of Haematology, Haemostasis and Oncology, Hannover Medical School, Hannover, Germany; 4 MRC-University of Glasgow Centre for Virus Research, Glasgow, UK; 5 Institute for Medical Virology and Epidemiology, University Hospital Tuebingen, Elfriede-Aulhorn-Str. 6, 72076 Tuebingen, Germany; 6 Present Address: National Centre for Biological Sciences, Bangalore, India

**Keywords:** Haematopoietic stem cell transplantation, Human cytomegalovirus, High-throughput sequencing, Sequence diversity, Genotyping, Multiple-strain infection

## Abstract

Human cytomegalovirus (HCMV) is an important opportunistic pathogen in allogeneic haematopoietic stem cell transplant (HSCT) recipients. High-throughput sequencing of target-enriched libraries was performed to characterise the diversity of HCMV strains present in this high-risk group. Forty-four HCMV-DNA-positive plasma specimens (median viral input load 321 IU per library) collected at defined time points from 23 HSCT recipients within 80 days of transplantation were sequenced. The genotype distribution for 12 hypervariable HCMV genes and the number of HCMV strains present (i.e. single- vs. multiple-strain infection) were determined for 29 samples from 16 recipients. Multiple-strain infection was observed in seven of these 16 recipients, and five of these seven recipients had the donor (D)/recipient (R) HCMV-serostatus combination D + R + . A very broad range of genotypes was detected, with an intrahost composition that was generally stable over time. Multiple-strain infection was not associated with particular virological or clinical features, such as altered levels or duration of antigenaemia, development of acute graft-versus-host disease or increased mortality. In conclusion, despite relatively low viral plasma loads, a high frequency of multiple-strain HCMV infection and a high strain complexity were demonstrated in systematically collected clinical samples from this cohort early after HSCT. However, robust evaluation of the pathogenic role of intrahost viral diversity and multiple-strain infection will require studies enrolling larger numbers of recipients.

**Supplementary Information:**

The online version contains supplementary material available at 10.1007/s00430-021-00722-5.

## Introduction

Despite excellent screening strategies and antiviral treatment regimens applied prophylactically or pre-emptively, human cytomegalovirus (HCMV) remains a significant pathogen following allogeneic haematopoietic stem cell transplantation (HSCT) [1–4]. Primary infection, reinfection and reactivation can result in subclinical viraemia or systemic disease and end-organ manifestations. Moreover, HCMV is associated with an increased risk of acute graft-versus-host disease (aGvHD) as well as opportunistic infections by other pathogens [5–7]. One well-established risk factor for HCMV infection and related complications after HSCT is the HCMV immunoglobulin G serostatus of the donor (D) and recipient (R), with the highest risk for recipients with the D − R + combination because of delayed HCMV-specific immune reconstitution [8, 9].

HCMV (species *Human betaherpesvirus 5)* has a double-stranded DNA genome of 236 kbp that contains at least 170 open reading frames (ORFs) encoding functional proteins [10]. The diversity of HCMV strains is particularly apparent in a subset of hypervariable genes, each of which presents several stable genotypes, and interstrain recombination during HCMV evolution has generated huge numbers of strains worldwide [11–16]. In addition, reactivation of multiple latent HCMV strains as well as exogenous reinfection with one or more strains can contribute to viral diversity within individuals [17–19]. Prior to the advent of high-throughput sequencing (HTS), many genotyping studies based on polymerase chain reaction (PCR) and Sanger sequencing of single hypervariable genes were aimed at linking clinical outcome parameters to aspects of viral diversity, but were compromised by limitations in understanding strain complexity within individuals [20–24].

In recent years, several studies have used HTS to analyse diversity across the whole HCMV genome [11, 12, 25]. Target capture approaches, which enrich the viral sequences in a sample by hybridisation to bait libraries, have provided further advances, making it possible to generate HCMV sequence data directly from a variety of HCMV-DNA-positive diagnostic specimens. This relatively unbiased approach avoids unnecessary PCR amplification steps or the need to isolate strains in cell culture [14, 19, 26, 27]. In a previous study, we observed that HCMV-DNAaemia was linked to different patterns of viral population dynamics in a retrospectively assembled collection of clinical specimens from HCMV-positive immunocompromised recipients [28]. However, to diminish potential bias due to selection of cases (e.g. by preferentially choosing recipients with high viral loads) and thus to analyse the extent of HCMV genome diversity and its potential impact on clinical outcome more robustly, investigations on a prospective and homogeneous cohort are desirable.

Here, we present HTS-generated data on HCMV populations from plasma specimens collected at defined time points from a clinically well-characterised HSCT cohort, focusing on viral intrahost diversity and strain complexity.

## Materials and methods

### Study design and sample collection

Plasma specimens (*n* = 253) were obtained prospectively from a cohort of HSCT recipients (*n* = 42) who had been transplanted at Hannover Medical School and had the following serostatus combinations: D − R + (n = 14), D + R + (n = 22) and D − R − (*n* = 6). Three to seven samples were collected from each recipient within defined time periods (7–10, 11–17, 18–24, 25–31, 32–38, 47–53 and 77–83 days post-transplantation) and stored at − 20 °C. All specimens with detectable HCMV-DNAaemia were sequenced. They were designated by the patient number and day of sampling. To simplify the nomenclature, specimens taken during the sampling periods were designated as originating from day 8, 14, 21, 28, 35, 50 or 80, respectively, e.g. 1001–35 signifying plasma obtained in the period around day 35 from patient 1001. Statistical analyses were conducted to investigate correlations between viral sequence features (i.e. presence of multiple strains) and the following clinical features, which were retrieved from the medical records: presence of aGvHD, T cell depletion, subsequent survival and number and duration of HCMV antigenaemia episodes. This study was approved by the institutional review board of Hannover Medical School (no. 2527-2014 and no. 2906-2016).

### Clinical procedures and definitions

Regular (i.e. at least weekly) HCMV screening of recipients after transplantation was based on HCMV pp65 antigenaemia testing [29] according to the standard procedures being applied in the HSCT unit of Hannover Medical School at that time. Plasma samples utilised in the present study were collected independently of samples used for antigenaemia monitoring. HCMV reactivation or infection was defined as ≥ 5 pp65 antigen-positive cells per 4 × 10^5^ leukocytes or ≥ 2 pp65 antigen-positive cells per 4 × 10^5^ leukocytes in two or more consecutive samples, and was treated pre-emptively with antiviral drugs at the physicians’ discretion. For statistical purposes, the end of an antigenaemia episode was defined as the point at which at least one negative HCMV pp65 antigenaemia result had been obtained; HCMV pp65 antigenaemia values were considered until day 100 for this study. Diagnosis of aGvHD was based on previously established criteria [30]. T cell depletion in vivo was assumed if antithymocyte globulin (ATG) had been part of the conditioning regimen. Overall survival was followed up as long as recipients attended outpatient clinic visits.

### Statistics

Pearson’s Chi-square-test or Fisher’s exact test was applied to determine the statistical significance of differences between groups (i.e. multiple-strain vs. single-strain infection), using SPSS statistics version 25 (IBM). The Mann–Whitney test was performed to compare median values (e.g. viral loads or antigenaemia levels) among groups, using GraphPad Prism version 7.03 (GraphPad Software). Significance was indicated by *p* values of < 0.05.

### DNA extraction and viral load quantification

DNA extraction and quantitative HCMV real-time PCR were carried out as described previously to identify HCMV-DNA-positive plasma samples and quantify viral loads [31]. Calibration was carried out according to the HCMV WHO standard [32], with a lower limit of linear quantification of 1000 IU/ml. HCMV-positive samples with loads below this limit were assigned the extrapolated viral load value calculated by the PCR cycler (ABI 7500) software. The input number of HCMV genomes used to prepare sequencing libraries was calculated in IU (approximately equivalent to genome copies).

### High-throughput sequencing (HTS)

Library preparation and sequencing were performed as described previously with a few modifications [14, 26, 28, 33]. Briefly, DNA was sheared, sequencing libraries were prepared using a library preparation kit (KAPA Biosystems) and PCR pre-amplification (6–14 cycles) was conducted using adapter-specific primers. Up to 750 ng amplified DNA was target-enriched using custom HCMV-specific RNA baits (SureSelect XT kit, Agilent). HCMV-enriched libraries were indexed, amplified (17–20 cycles), multiplexed and sequenced on a MiSeq (Illumina) using 600v3 reagent kits to generate 2 × 300 nt paired-end reads.

### Assembly and analysis of HCMV genome sequences

De novo assembly of reads and generation of HCMV consensus genomes was performed as described previously [28], utilising a pipeline, including fastp (https://github.com/OpenGene/fastp) for adapter trimming, bowtie2 (http://bowtie-bio.sourceforge.net/bowtie2/index.shtml) for human read filtering, SPAdes (https://cab.spbu.ru/software/spades/) for contig assembly and CLC Genomics Workbench (CLC version 10.0.1) for contig scaffolding to the reference HCMV strain Merlin. The resulting draft genomes were trimmed of the inverted repeats at the HCMV genome termini (TR_L_ and TR_S_) and manually annotated from the Merlin strain reference. Comparison and visualisation of complete sequences were performed using BioEdit (https://bioedit.software.informer.com/7.2/) version 7.2.0.

### Genotyping and strain enumeration using motif read matching

To distinguish single-strain from multiple-strain infections and to monitor the composition of HCMV populations in individuals longitudinally, we used the previously established approach of motif read matching [14]. This exploits 12 HCMV genes (RL5A, RL6, RL12, RL13, UL1, UL9, UL11, UL73, UL74, UL120, UL146 and UL139), each of which exhibits a high level of nucleotide diversity and falls into several genotypes that may be monitored by searching for conserved motifs specific to each genotype. The numbers of reads containing these motifs (20–24 nucleotides (nt)) were determined for each of the 12 hypervariable genes by screening the data sets as described previously (https://centre-for-virus-research.github.io/VATK/HCMV_pipeline [14]). The number of HCMV strains represented in each data set was then calculated as the maximum number of genotypes detected for ≥ 2 genes, with a requirement for ≥ 10 reads to contain the relevant motifs and for these to represent ≥ 2 % of the reads identified for all genotypes of the relevant gene. Genotype abundance was visualised in R (https://www.r-project.org) using ggplot2 3.3.0 (https://ggplot2.tidyverse.org).

### Variant analyses

Analysis of intrahost HCMV genome variation was performed as described previously [28]. Duplicate reads from identical HCMV-DNA fragments were removed from each data set (deduplicated) using Picard v2.3.0. Briefly, variants (single nucleotide polymorphisms (SNPs)) were called after mapping the deduplicated reads from each sample to the consensus sequence derived from the same sample or, in case of data from longitudinal samples, to the consensus sequence derived from the initial sample. Valid variants were defined by the following criteria in deduplicated data sets: read depth ≥ 25, average base call quality ≥ 20, forward/reverse read balance 0.3–0.5 and variant frequency ≥ 2 %.

### Data deposition

Data sets were purged of human reads and deposited in the European Nucleotide Archive under accession numbers ERX4241108–ERX4241138. HCMV genome sequences from recipients infected by single strains were deposited in GenBank under accession numbers MT649468–MT649474.

## Results

### HTS and data set quality

All plasma samples with detectable HCMV loads (*n* = 44), obtained from 23 HSCT recipients, were sequenced (Table S1). The median viral input load was 321 IU per library (range 33–155,000). Data sets meeting the quality criteria for genotyping and strain enumeration were obtained from 24 samples (median input 863 IU per library, range 135–155,000) from 14 HSCT recipients. De novo assembly of consensus sequences was achieved for these data sets, with a median average coverage depth of 836 reads/nt (range 103–5925) for all reads and 58 reads/nt (range 11–4624) after deduplication (Table S1). An additional five data sets (median input viral load 221 IU per library) did not meet the quality criteria or yield consensus sequences by de novo assembly, but contained sufficient data for partial genotyping and were retained for further analysis, bringing the number of samples analysed to 29 and the number of recipients represented to 16 (D − R + , *n* = 7, and D + R + , *n* = 9). The clinical and virological characteristics of the HSCT recipients included in the analysis are shown in Table 1. The remaining 15 data sets were excluded because of failure to produce a sequencing library or very poor data quality. These were obtained from samples with viral loads below the limit of quantification of the HCMV-PCR, with a median input of 131 IU per library (range 33–337).

**Table 1:** Clinical and virological characteristics of HSCT recipients yielding analysed sequence data

Recipient no.	Age (years)	Sex	Diagnosis	Samples (no.)	HCMV serostatus	Multiple-strain infection (no. of strains)	In vivo T cell depletion	Survival (days after HSCT; cause of death)	aGvHD (grade)	DNAaemia at day 80	HCMV reactivation episodes (no.)^b^	Antiviral treatment
1001	48	F	MM	1	D + R +	No	No	Alive (2513)	Yes (II)	No	1	Valganciclovir
1002	19	F	NHL	1	DR +	Yes (2)	Yes	Alive (2484)	No	No	1	Valganciclovir
1011^a^	24	F	AA	1	D + R +	No	Yes	Alive (2254)	Yes (II)	Yes	0^c^	None
1012	50	M	MDS	4	D + R +	Yes (2)	Yes	Alive (2397)	Yes (II)	No	1	Cidofovir
1020	51	F	CML	1	D + R +	No	No	Deceased (211; pneumonia)	Yes (II)	Yes	1	Valganciclovir
1028	57	F	AML	2	D − R +	Yes (2)	Yes	Deceased (711; relapse)	No	No	1	Valganciclovir
1032^a^	54	M	AML	2	D + R +	Yes (3)	Yes	Deceased (134; multiorgan failure)	Yes (II)	No	2	Ganciclovir, foscarnet
1033	43	M	AML	2	D – R +	No	Yes	Alive (2164)	Yes	No	2	Valganciclovir
1036	63	F	MM	2	D – R +	No	Yes	Deceased (254; progress of disease)	No	Yes	2	Ganciclovir, cidofovir
1040	62	M	AML	1	D + R +	Yes (2)	Yes	Alive (1849)	No	No	1	Valganciclovir
1041	27	M	MDS	2	D − R +	No	Yes	Alive (1829)	Yes (II/III)	Yes	1	Valganciclovir
1047	63	M	AML	2	D − R +	No	Yes	Alive (1755)	No	Yes	3	(Val)ganciclovir, foscarnet
1048^a^	53	M	AML	3	D − R +	No	Yes	Deceased (1445; relapse, pneumonia)	No	No	2	Foscarnet, cidofovir
1065	59	F	CLL	1	D + R +	Yes (2)	No	Alive (1740)	Yes (I-II)	No	1	Valganciclovir
1069	54	M	AML	2	D + R +	Yes (2)	Yes	Alive (1621)	No	No	1	Valganciclovir
1074^a^	58	M	AML	2	D + R +	No	Yes	Alive (1281)	No	No	1	Valganciclovir

*AA* aplastic anaemia, *aGvHD* acute graft-versus-host disease, *AML* acute myeloid leukaemia, *CLL* chronic lymphatic leukaemia, *CML* chronic myeloid leukaemia, *D* donor, *MM* multiple myeloma, *HSCT* haematopoietic stem cell transplantation, *MDS* myelodysplastic syndrome, *NHL* non-Hodgkin’s lymphoma, *R* recipient

^a^Sequence data from some samples did not meet the quality criteria but were analysed nonetheless

^b^Based on HCMV antigenaemia data collected until day 100

^c^No detectable HCMV antigenaemia

### Genotyping and strain enumeration

**Fig. 1:**
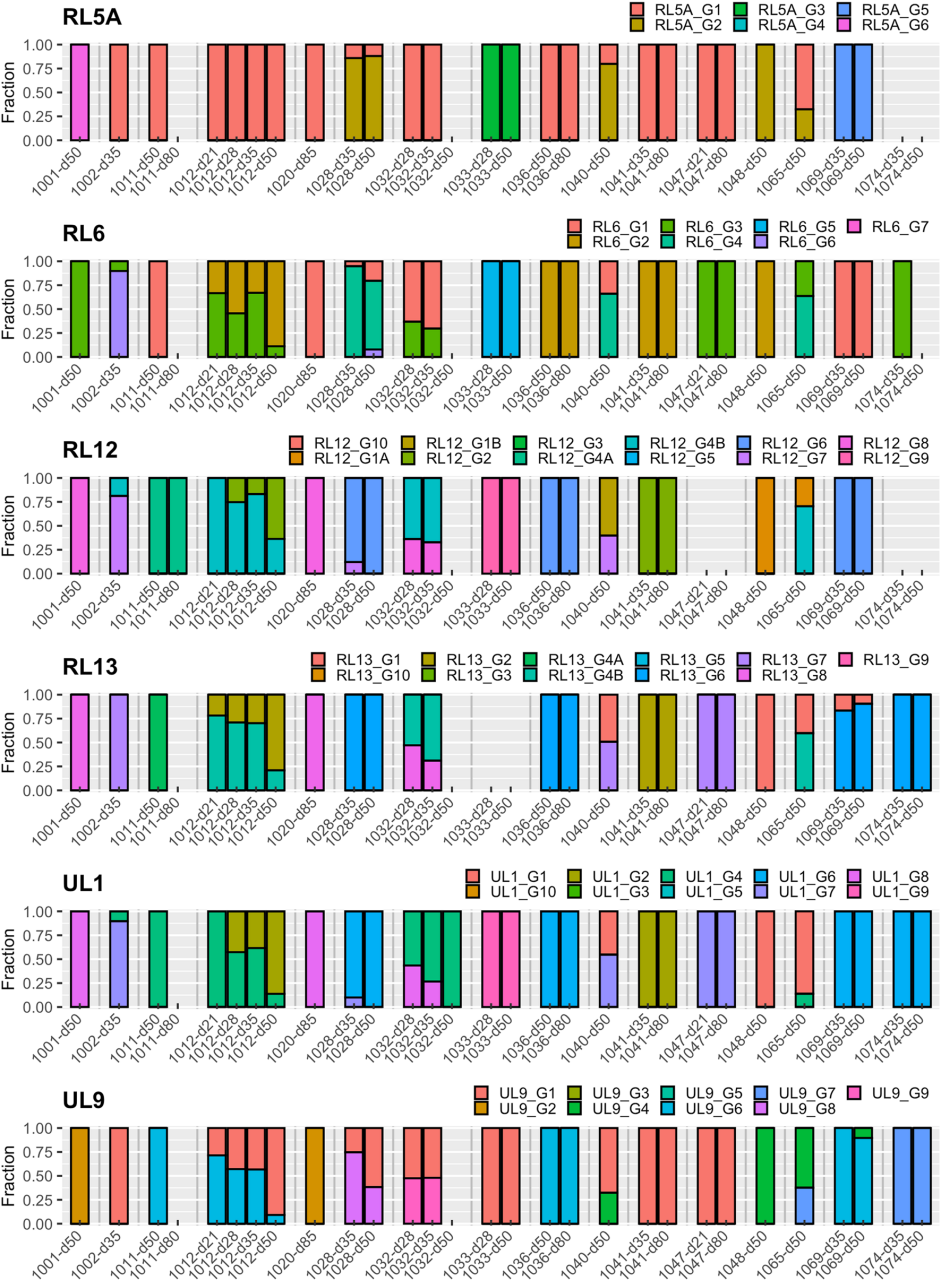

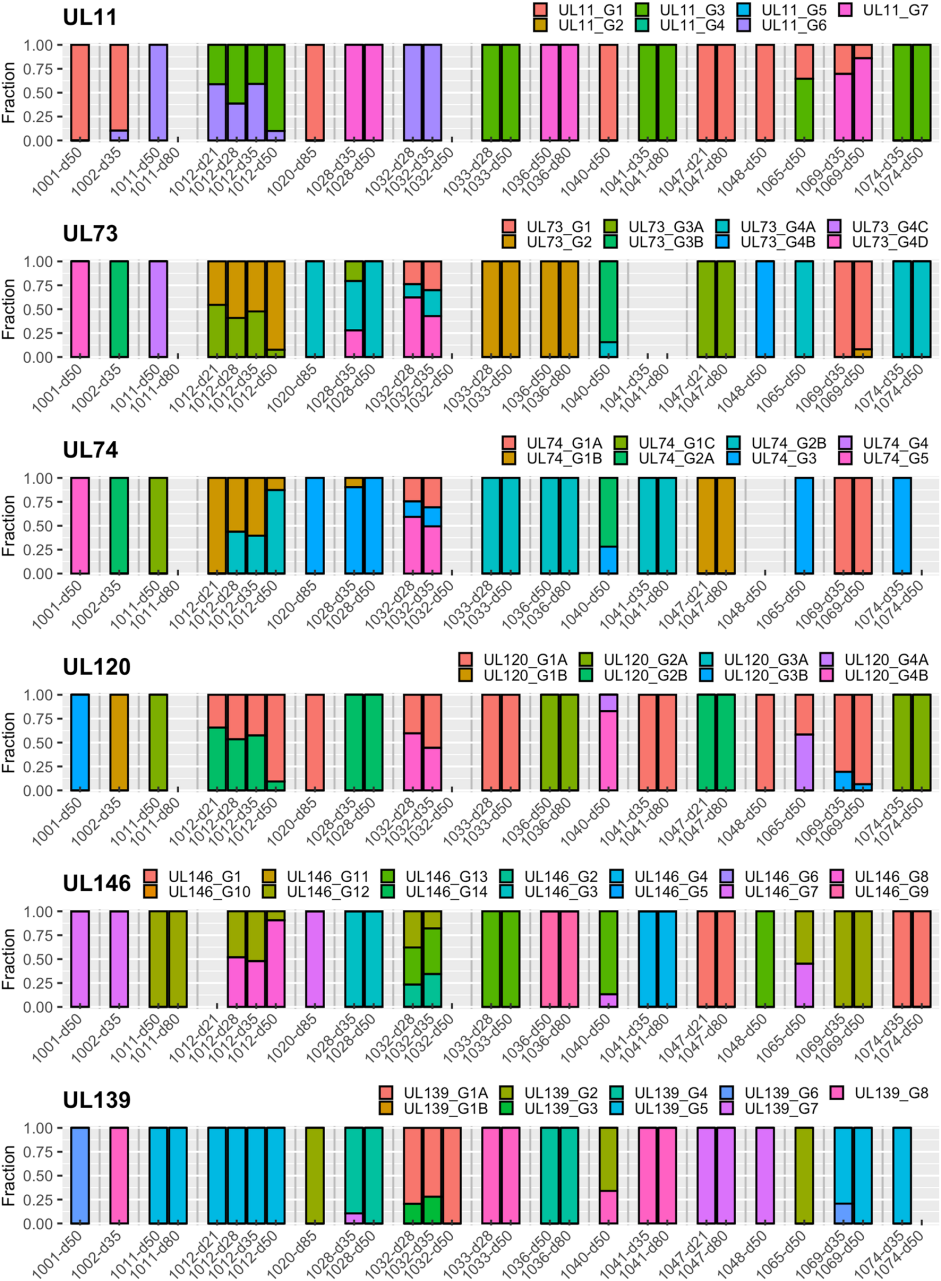
Genotype profile of hypervariable genes. Genotype profile and relative abundance of HCMV genes RL5A–UL139 genotypes in the data sets obtained from 29 plasma samples from 16 HSCT recipients. Each colour represents a different genotype of a hypervariable gene. Each bar represents a different sample, grouped by subject. The Y-axis indicates the fraction of genotypes in the total sequence reads for that gene in the sample. Underlying data are shown in Table S1. The genotype scoring criteria are described in Materials and Methods

Genotyping was carried out by counting reads containing genotype-specific motifs in 12 hypervariable HCMV genes as described above. In total, 7/16 recipients (D + R + (*n* = 5) and D − R + (*n* = 2)) had multiple-strain infections involving two or three HCMV strains (Table 1). Genotyping data were available for a subset of the 12 genes for the five samples that did not meet the quality criteria: three genes for samples 1011–80 and 1032–50 (samples from other time points from these recipients yielded data for the full range of hypervariable genes), 11 genes for 1048–50 and 1074–35 and seven genes for 1074–50. A very wide range of genotypes was detected in the 12 genes analysed (Table 2). The composition of genotypes was generally stable among longitudinal samples from the same recipient during the observed period, although the relative abundance of genotypes in multiple-strain infection varied (Fig. 1), and in one case (recipient 1012) there was a switch of the dominant population over time. Detailed read numbers for each genotype-specific motif are presented in Table S1.

**Table 2:** Genotype analysis and strain enumeration

Data set	Strain number^b^	Genotypes^a^											
		RL5A	RL6	RL12	RL13	UL1	UL9	UL11	UL73	UL74	UL120	UL146	UL139
1001–50	1	6	3	8	8	8	2	1	4D	5	3B	7	6
1002–35	2	1	3, 6	4B, 7	7	4, 7	1	1, 6	3B	2A	1B	7	8
1011–50	1	1	1	4A	4A	4	6	6	4C	1C	2A	12	5
1011–80^c^	1	–	–	4A	–	–	–	–	–	–	–	12	5
1012–21	2	1	2, 3	4B	2, 4B	4	1, 6	3, 6	2, 3A	1B	1A, 2B	8, 12	5
1012–28	2	1	2, 3	2, 4B	2, 4B	2, 4	1, 6	3, 6	2, 3A	1B, 2B	1A, 2B	8, 12	5
1012–35	2	1	2, 3	2, 4B	2, 4B	2, 4	1, 6	3, 6	2, 3A	1B, 2B	1A, 2B	8, 12	5
1012–50	2	1	2, 3	2, 4B	2, 4B	2, 4	1, 6	3, 6	2, 3A	1B, 2B	1A, 2B	8, 12	5
1020–80	1	1	1	8	8	8	2	1	4A	3	1A	7	2
1028–35	2	1, 2	1, 4	6, 7	6	6, 7	1, 8	7	3A, 4A, 4D	1B, 3	2B	3	4, 7
1028–50	2	1, 2	1, 4, 6	6	6	6	1, 8	7	4A	3	2B	3	4
1032–28	3	1	1, 3	4B, 6, 8	4B, 8	4, 8	1, 9	6	1, 4A, 4D	1A, 3, 5	1A, 4B	2, 12, 13	1A, 3
1032–35	3	1	1, 3	4B, 8	4B, 8	4, 6, 8	1, 9	6, 7	1, 3B, 4A, 4D	1A, 3, 5	1A, 4B	2, 12, 13	1A, 3
1032–50^c^	1	–	–	–	–	4	–	–	1	–	–	–	1A
1033–28	1	3	5	9	–	9	1	3	2	2B	1A	13	8
1033–50	1	3	5	9	–	4, 9	1	3	2	2B	1A	13	8
1036–50	1	1	2	6	6	6	6	7	2	2B	2A	9	4
1036–80	1	1	2	6	6	6	6	7	2	2B	2A	9	4
1040–50	2	1, 2	1, 4	1B, 7	1, 7	1, 7	1, 4	1	3B, 4A	2A, 3	4A, 4B	7, 13	2, 8
1041–35	1	1	2	2	2	2	1	3	–	2B	1A	4	8
1041–80	1	1	2	2	2	2	1	3	–	2B	1A	4	8
1047–21	1	1	3	–	7	7	1	1	3A	1B	2B	1	7
1047–80	1	1	3	–	7	7	1	1	3A	1B	2B	1	7
1048–50^c^	1	2	2	1A	1	1	4	1	3B, 4B	–	1A	13	7
1065–50	2	1, 2	3, 4	1A, 4B	1, 4B	1, 4	4, 7	1, 3	4A	1A, 3	1A, 4A	7, 12	2, 5
1069–35	2	5	1, 5	1A, 6	1, 6	1, 6	6	1, 7	1, 2	1A	1A, 3B	12	5, 6
1069–50	2	3, 5	1	1A, 6	1, 6	1, 6	4, 6	1, 7	1, 2	1A, 2B	1A, 3B	8, 12	5, 6
1074–35^c^	1	–	3	–	6	6	7	3	4A	3	2A	1	5
1074–50^c^	1	–	–	–	6	6	7	3	4A	–	2A	1	–

– Indicates insufficient reads meeting the threshold for genotyping that gene

^a^Genotypes assigned to the 29 data sets were determined using motifs for 12 hypervariable HCMV genes

^b^The number of strains detected in a data set was calculated by the maximum number of genotypes detected for at least two genes according to the definitions described in Materials and Methods. Discrepant genotype numbers among different time points in certain genes of a single patient (e.g. UL1 or UL139 in patient 1032) do not necessarily mean that these genotypes emerged de novo or disappeared, but rather were already present in both samples, although in some cases below the cut–off level used to define multiple–strain reactivation. Detailed read numbers for genotype motifs are presented in Table S1

^c^Samples that did not meet the quality criteria for assembly, but yielded genotype data. Interpretations with regard to multiple–strain infections are limited due to relatively low sequencing coverage and should be viewed in the context of other data sets from the same individual

### Variant analysis

**Fig. 2:**
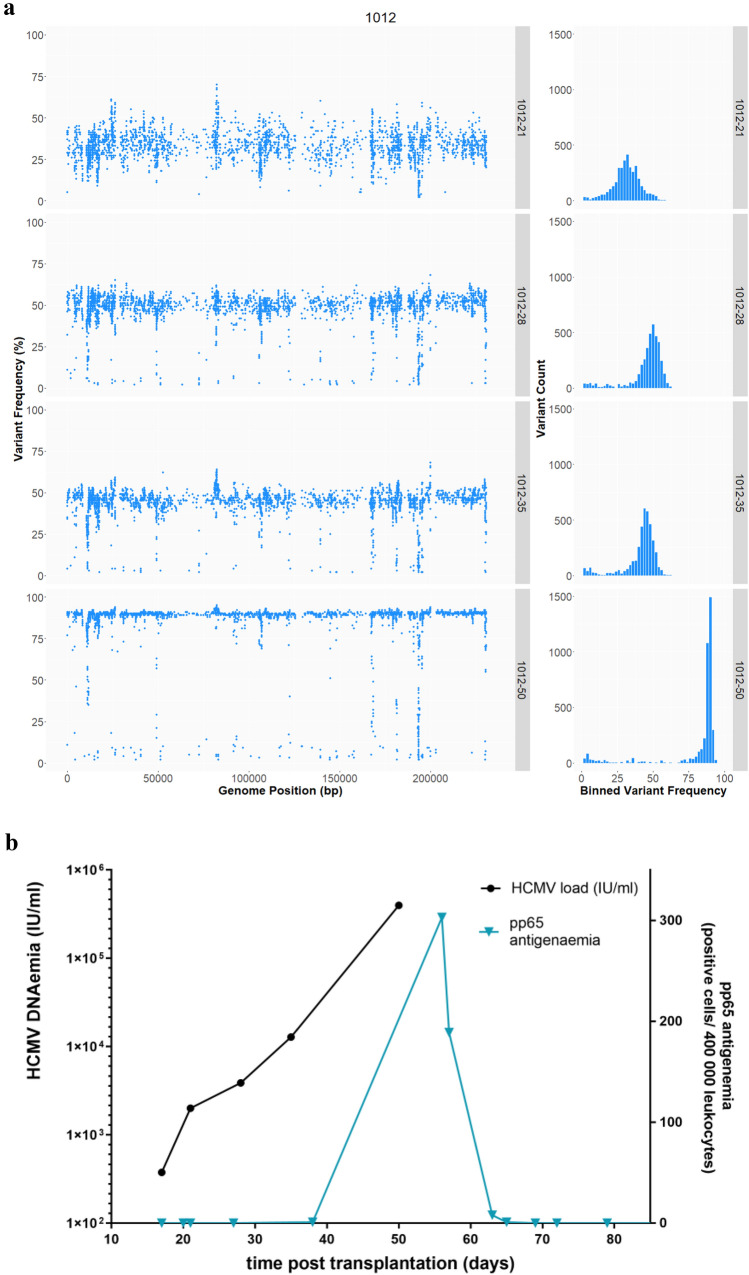
Longitudinal HCMV sequence diversity in recipient 1012. **a** Variant analysis was conducted in relation to the consensus sequence from day 21. The frequency of variants (Y-axis) was plotted against the position on the genome (X-axis). The histogram on the left shows the variants binned according to their frequency percentage (X-axis) plotted against the absolute number of variants (Y-axis). **b** HCMV DNA load (IU/ml, left Y-axis) and pp65 antigenaemia (positive cells/400,000 leukocytes, right Y-axis) in plasma were plotted against time after transplantation (days, X-axis). Samples from four time points (days 21, 28, 35 and 50 post-transplantation) were sequenced

Variant analysis was conducted for data sets having a mean coverage depth of ≥ 25 deduplicated reads/nt in order to detect minor populations. This analysis (Figure S1) confirmed the distinction made between recipients with single- or multiple-strain infection by genotyping, and also revealed the population dynamics within recipient 1012 (Fig. 2). For this patient, multiple populations were already detectable at the first time point (day 21), with 3174 intrahost variants present at a median frequency of 32 %. At the last time point (day 50), the median frequency of intrahost variants called against the consensus sequence from the initial time point increased to 89 % (Fig. 2a). DNAaemia levels increased with the proportion of the variant population (Fig. 2b).

### Association of HCMV diversity with clinical features

Statistical analyses of the clinical and viral diversity features of the 16 recipients with sufficient HTS data indicated that multiple-strain infection was associated with the absence of DNAaemia at day 80 post-transplantation (*p* = 0.034) (Table 3). However, multiple-strain infection was not associated with antigenaemia duration or level, peak viral load, serostatus of the donor or recipient, in vivo T cell depletion, development of aGvHD, or fatal outcome (Table 3). Statistical tests for associations between the specific genotype of any of the 12 hypervariable genes with clinical features were not conducted because of the large number of genotypes detected and the relatively small cohort size.

**Table 3:** Comparison of clinical and virological parameters between recipients infected with single or multiple HCMV strains

	Recipients with single-strain infection (*n* = 9)	Recipients with multiple-strain infection (*n* = 7)	*p* value
Sex male, *n* (%)	5 (56)	4 (57)	1
Mean age (range), years	51 (24–63)	51 (19–62)	0.633
Donor/recipient HCMV serostatus, *n* (%)			0.358
D + R +	4 (44)	5 (71)	
D − R +	5 (56)	2 (29)	
In vivo T cell depletion, *n* (%)	7 (78)	6 (85)	1
aGvHD, *n* (%)	5 (56)	3 (42)	1
Median time to onset of aGvHD (range), days after HSCT	35 (10–66)	36 (28–47)	0.786
Fatal outcome, *n* (%)	3 (33)	2 (29)	1
Median time to fatal outcome (range), days after HSCT	254 (211–1445)	423 (134–711)	0.8
More than one reactivation episode, *n* (%)	4 (44)	1 (14)	0.308
DNAaemia detected at day 80, *n* (%)	5 (56)	0 (0)	**0.034**
Median HCMV load (range), IU/ml plasma^a^	1680 (303–5790)	2010 (348–398,000)	0.374
Median viral load peak value (range), IU/ml	2550 (463–5790)	1630 (692–398,000)	0.918
Median duration of individual antigenaemia episodes (range), days after HSCT	15 (0–38)	11 (7–19)	0.202
Median duration of overall HCMV-antigenaemia (range), days	25 (0–49)	14 (9–22)	0.142
Median antigenaemia peak value (range), pp65 positive cells/400 000 leukocytes	33 (0–400)	88 (13–303)	0.837

Bold value indicates statistically significant *p* values of < 0.05

*D* donor, *aGvHD* acute graft-versus-host disease, *HSCT* haematopoietic stem cell transplantationm, *R* recipient

^a^For all plasma samples with sequence data of the respective group

## Discussion

Target enrichment protocols combined with HTS have been utilised successfully to investigate the strain complexity, population dynamics and antiviral resistance of HCMV directly in human subjects [14, 26–28, 34]. These studies have generally involved heterogeneous retrospective cohorts. In contrast, the present study focused on a cohort of HSCT recipients from whom plasma samples were collected prospectively at defined time points during a period of 80 days after transplantation. However, probably due to efficient clinical management and pre-emptive therapy, viral loads were generally low in the recipients identified as HCMV positive. Nevertheless, data sets worthy of analysis could be obtained from 29 samples originating from 16 recipients.

Infection with multiple HCMV strains was detected frequently, in nearly half (44 %) of the recipients (*n* = 5 D + R + and *n* = 2 D − R +). PCR-based genotyping studies have previously implied the presence of multiple strains in solid organ and HSCT recipients and bioinformatic analyses of HTS data have shown previously that the presence of multiple strains is the major source of HCMV intrahost diversity [14, 17, 19, 35–37]. A study using HTS to genotype genes UL73 and UL74 (which are adjacent and therefore linked genotypically) reported a similar rate of multiple genotypes in 18/29 (62 %) of successfully genotyped specimens from HSCT recipients with active episodes of HCMV infection [38]. The fact that multiple-strain infection may originate from reactivation of multiple strains in the latent repository of a seropositive recipient or from the graft from a seropositive donor may explain the high frequency of such infection in D + R + recipients. For the two D − R + patients, we speculate that multiple-strain infections may have derived from the latent HCMV repository. As our recent study [37] on HCMV populations in breast milk revealed that approximately half (*n* = 8/15) of immunocompetent HCMV-seropositive women reactivated multiple HCMV strains upon lactation, the presence of multiple, latent HCMV strains in adults and thus also in HSCT recipients may be common. Other rare transmission sources for de novo HCMV re-infection, such as blood transfusions, HCMV-shedding household contacts (e.g. infants) or nosocomial exposure, cannot be ruled out, but seem rather unlikely due the use of leucocyte-depleted, HCMV-negative blood products and strict hygienic precautions.

In our study, genotyping of hypervariable genes detected a remarkable variety of genotypes that remained stable over the observed time period in longitudinal samples from the same individual, and yet no two recipients had identical combinations of genotypes, consistent with the view that interstrain recombination has generated enormous numbers of strains during HCMV evolution. Similarly, extensive HCMV strain variability among different individuals with high intrahost stability has also been reported for breast milk samples from European and African women in studies that also used genotyping of hypervariable genes to distinguish single-strain from multiple-strain infections [26, 37]. However, despite genotypic stability, the relative abundance of genotypes and the strains they represent may change over time in an individual, as observed in the present study. These findings are in line with our previous study of HCMV genomes in retrospectively collated sample sets of renal transplant and HSCT recipients, and also with other studies focused on a small number of hypervariable genes in lung transplant recipients [28, 39]. The complexity of HCMV populations in multiple-strain infection can fluctuate in a relatively short period of time, as observed in recipient 1012, in whom the dominant population switched within a time period of 29 days. This phenomenon illustrates how fast the strain repertoire can change in infected individuals, and is consistent with the previously reported short doubling time of HCMV [40].

Several studies have reported statistical trends associating the presence of multiple HCMV genotypes with higher peak load and longer duration of DNAaemia as well as slower viral clearance after transplantation [22, 36, 38, 41]. Some studies have also reported an association of multiple-strain infection with a worse clinical course, characterised, for example, by an increased number of episodes of HCMV disease, graft loss or opportunistic infection with other pathogens [17, 35]. However, other studies have not found such associations [41–43]. In the present work, multiple-strain infection was not significantly associated with prolonged duration or increased levels of antigenaemia or any other investigated clinical feature during the first 100 days after HSCT. In contrast, all seven recipients with multiple-strain infection had undetectable DNAaemia at 80 days post-infection (the last available sampling point) compared with 5/9 recipients with single-strain infection. This statistically slightly significant difference may have been due to the higher occurrence of multiple-strain infection in D + R + recipients, who have an overall decreased risk of severe or recurrent HCMV reactivation [8, 44]. HCMV-specific immune reconstitution occurs faster in recipients with a seropositive donor [9, 45], and recurrent reactivation occurred less often in R + D + recipients than in R + D − recipients in the present study, corroborating data obtained by others.

It is not clear whether and how multiple-strain HCMV infection influences viral load dynamics. The immune system may deal differently with antigenically different strains, leading to altered viral clearance properties in comparison with single-strain infection. In addition, complementation among strains or the contributions of viral factors with differing effects on virulence may enhance viral replication overall [38, 46]. However, even in a prospective study, higher viral loads or prolonged episodes of viraemia may make it easier technically to detect multiple HCMV strains without multiple-strain infection necessarily being associated with an inferior clinical outcome. In the present study, HTS analysis was performed without a preselection bias towards high viral loads, but sequencing failed for many samples with very low viral loads. This outcome led to a limitation of the present study, the relatively small number of samples from which HTS data sets of sufficient quality were generated. Although a substantial number (*n* = 253) of plasma samples were collected, the proportion that contained detectable levels of HCMV DNA was low (17 %), and generally low viral loads reduced the proportion of useable data sets even further (11 %). This challenge is likely to be encountered in future studies of this type, with major limitations set by both the type of clinical specimen and the concentration of HCMV DNA in the specimen. Nevertheless, the relatively low concentration of human DNA in plasma compared to whole blood and the application of an effective target enrichment protocol made it possible to obtain tractable sequence data sets from samples with an input of about ≥ 400 IU per library.

In order to investigate the key clinical questions more robustly, including the influence of multiple-strain infection or other viral markers on clinical outcome, it will be necessary to conduct large, prospective studies (e.g. studies in multicentric transplant recipient cohorts, which are currently being established) [47, 48]. In the HSCT setting, donor/recipient HCMV serostatus is an important factor in the risk and course of HCMV reactivation and disease, and this, as well as the incidence of multiple-strain infection, must be considered carefully in future studies. Development of more sensitive HTS protocols capable of sequencing the latent HCMV repository would also support efforts to elucidate HCMV population structure in both donor and recipient. HCMV strain composition differs greatly between individuals, and convincing associations between HCMV strain and clinical outcome have proven elusive in this cohort and in the literature generally. Investigations have also been conducted on the influence of the host side, i.e. of human genetic variants associated with HCMV reactivation and disease after HSCT. A recent study from Casto and colleagues identified a biologically convincing polymorphism that influences intracellular concentrations of immunosuppressive drugs after transplantation and reduces the risk of HCMV reactivation by 20 % [49]. This study also suggested that other, previously recognised variants mediating HCMV-related phenotypes do not significantly affect the risk of HCMV reactivation or disease after HSCT. These observations indicate that, if clinical associations with virus or host genotype exist, their contributions are unlikely to be strong individually, and that the predictive power of genotyping to identify virus or host biomarkers might be limited in comparison to other factors involved in HCMV pathogenesis.

## Supplementary Information

Below is the link to the electronic supplementary material.

Supplementary file1 (DOCX 292 KB)

Supplementary file2 (XLSX 39 KB)

## Data Availability

Data sets were purged of human reads and deposited in the European Nucleotide Archive (accession numbers ERX4241108–ERX4241138). Genome sequences from recipients infected by single strains were deposited in GenBank (MT649468–MT649474).

## Code availability

https://centre-for-virus-research.github.io/VATK/HCMV_pipeline
