# Supplementary material for: Human cytomegalovirus multiple-strain infections and viral population diversity in haematopoietic stem cell transplant recipients analysed by high-throughput sequencing
Source: Med Microbiol Immunol. 2021 Oct 6;210(5-6):291–304. doi: 10.1007/s00430-021-00722-5 (PMC8541999; doi:10.1007/s00430-021-00722-5)

**Supplementary Data**

**Table S1** **Summary on samples characteristics, basic HTS data and genotype analysis**

**Please see Excel file “S1”**

**Supplementary Figure S1 Variant analysis of data sets with a mean coverage depth of ≥25 for HCMV genome.** Intra-host variant frequency plots of all specimens yielding HCMV genomes with a mean coverage depth of ≥25 deduplicated reads/nt. Each dot in the left part of the plot represents an SNP with its corresponding position in the HCMV genome and its frequency. The right-side histogram shows the number of SNPs in each frequency bin. Variants were called by mapping the reads to the assembled HCMV genome. If samples from multiple time points were sequenced, reads were mapped against the first time point. This visualises multiple strains as bands of SNPs spanning the genome and allows the identification of shifting ratios between strains. For multiple-strain samples with similar abundance of constituent subpopulations, variants will appear above and below the 50% frequency threshold as assembly of a genome sequence clearly attributable to one of the two or three HCMV strains is not feasible (for example, this can be observed for individuals 1065 and 1040).


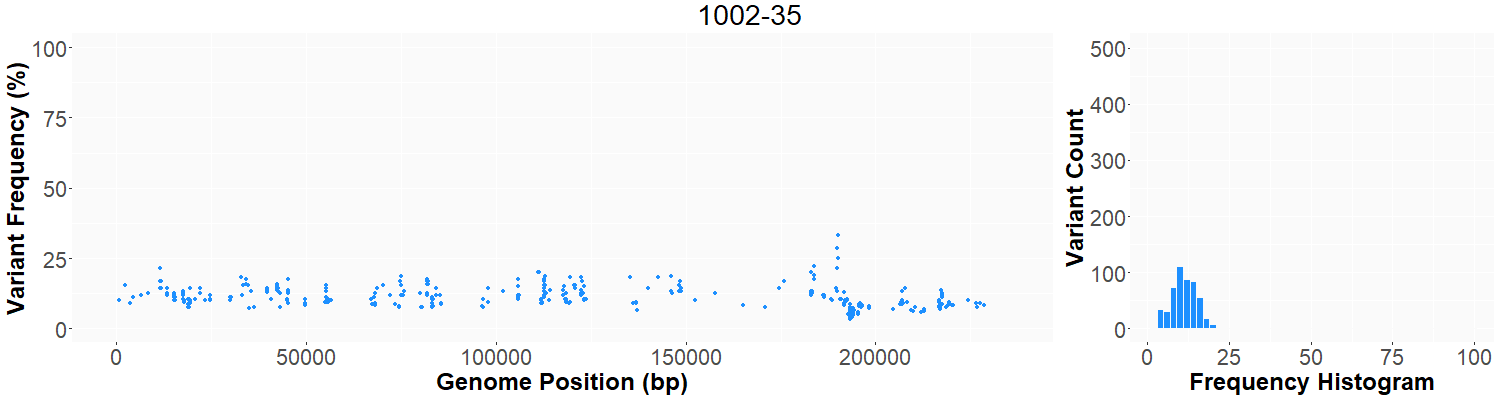

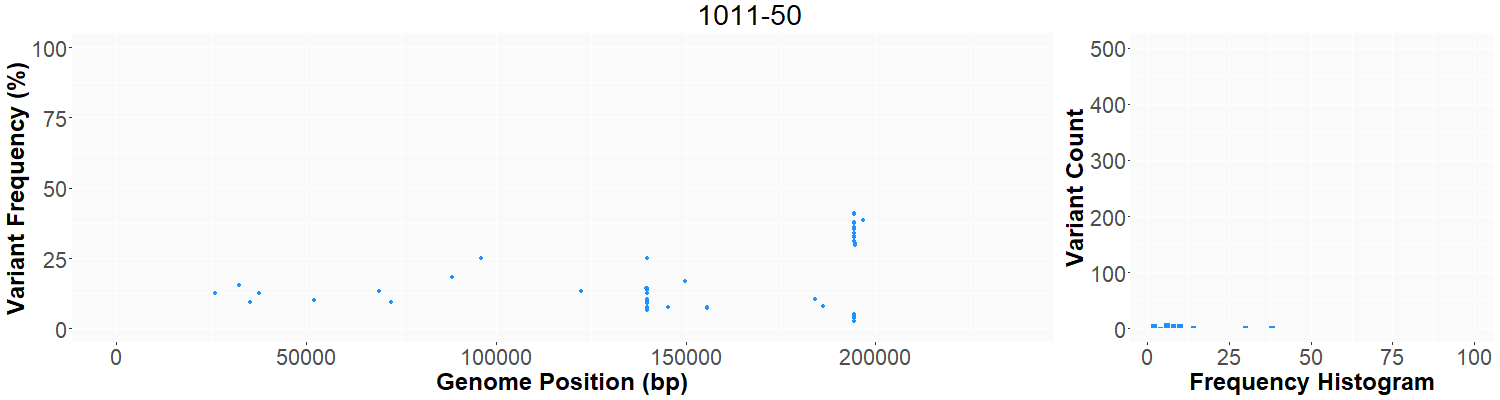

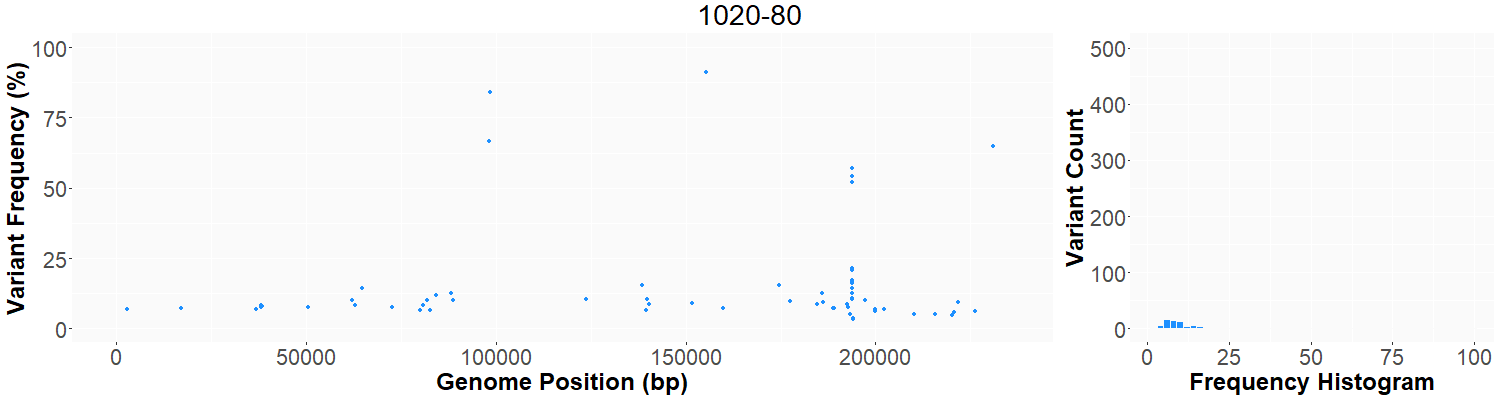

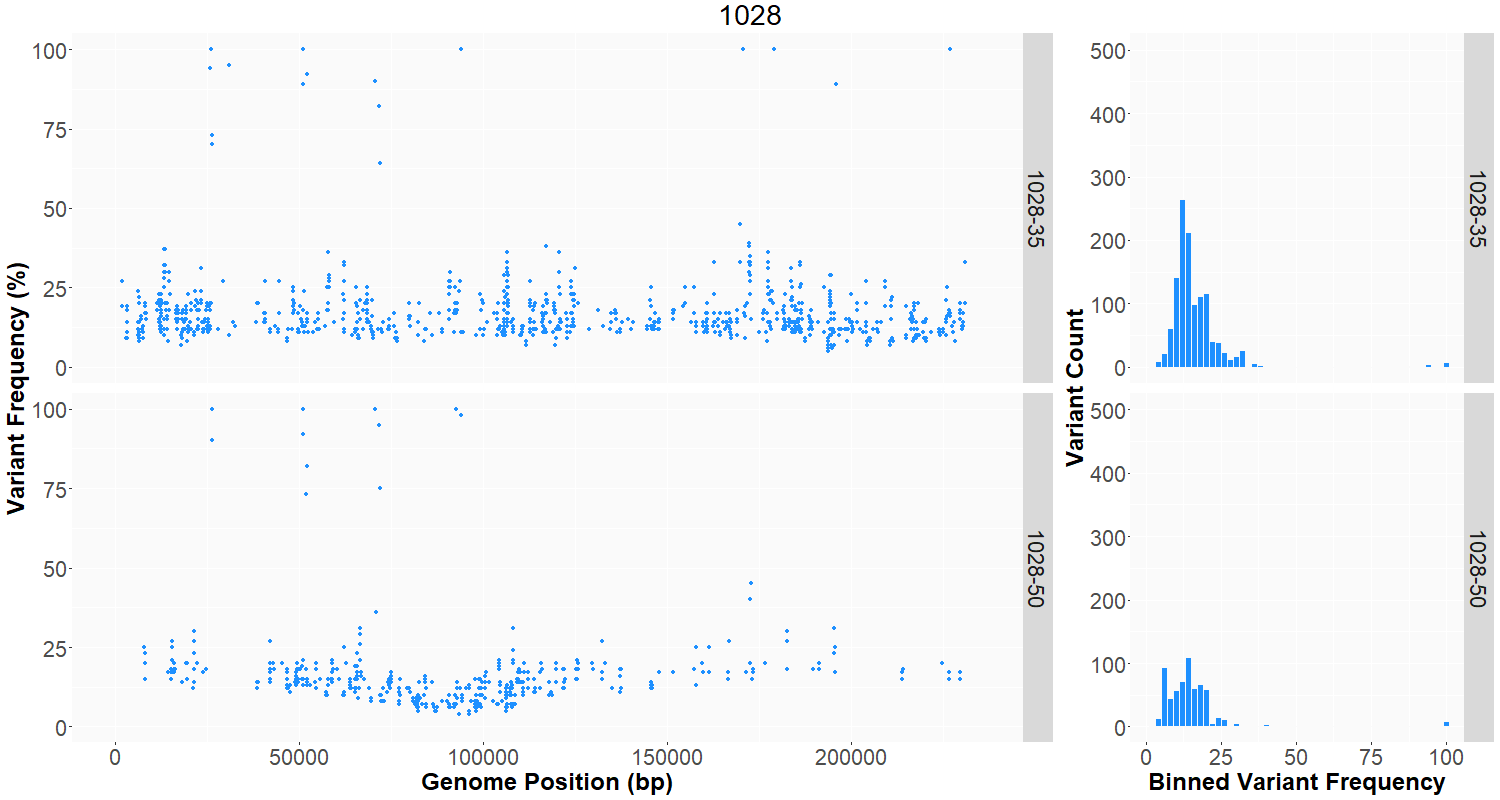

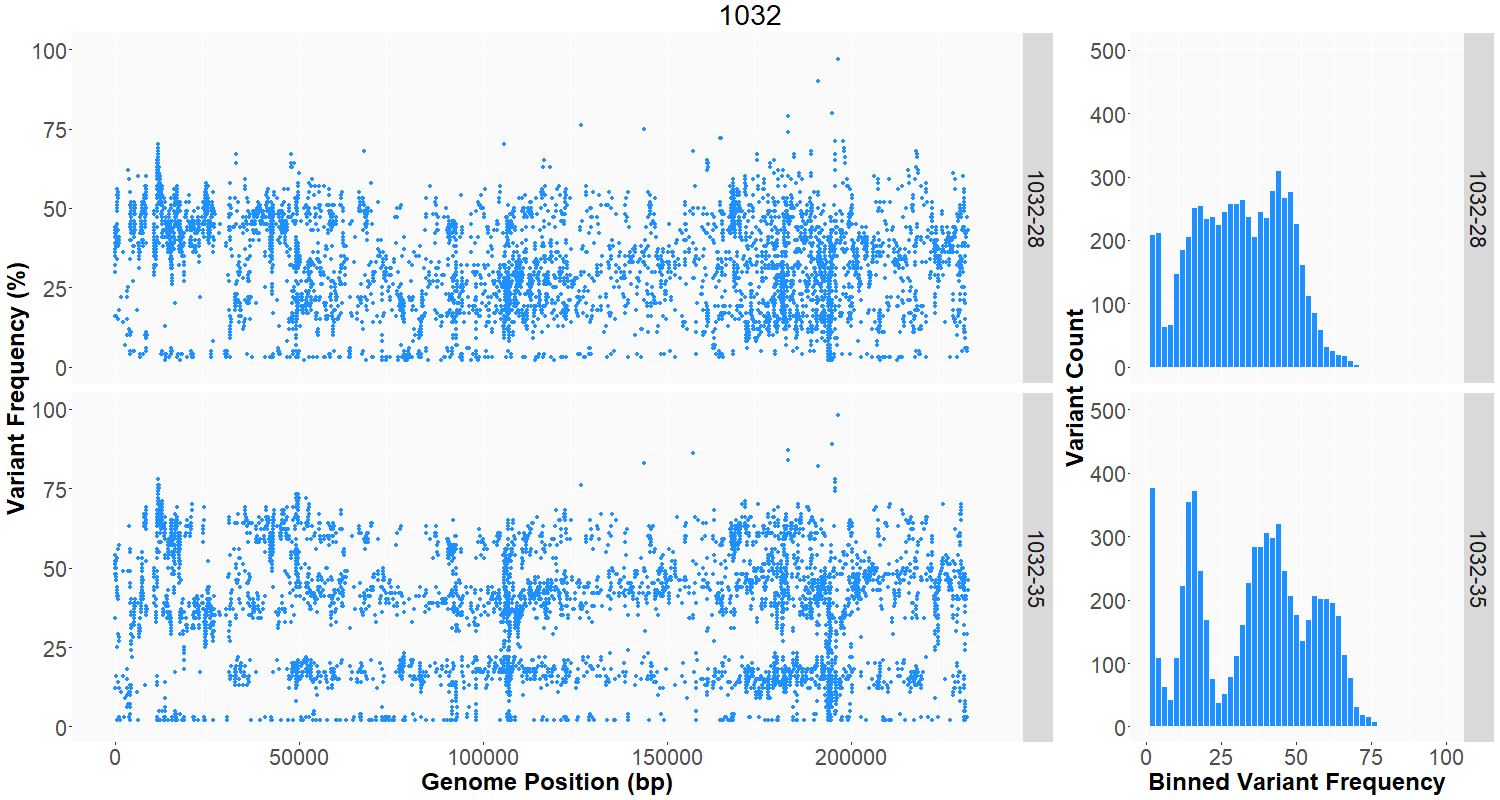

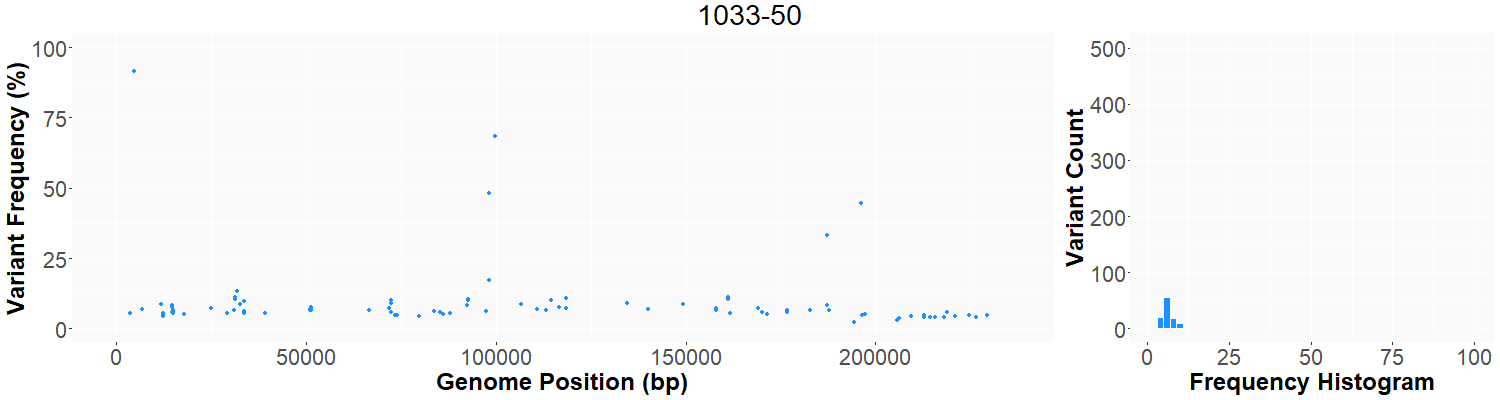

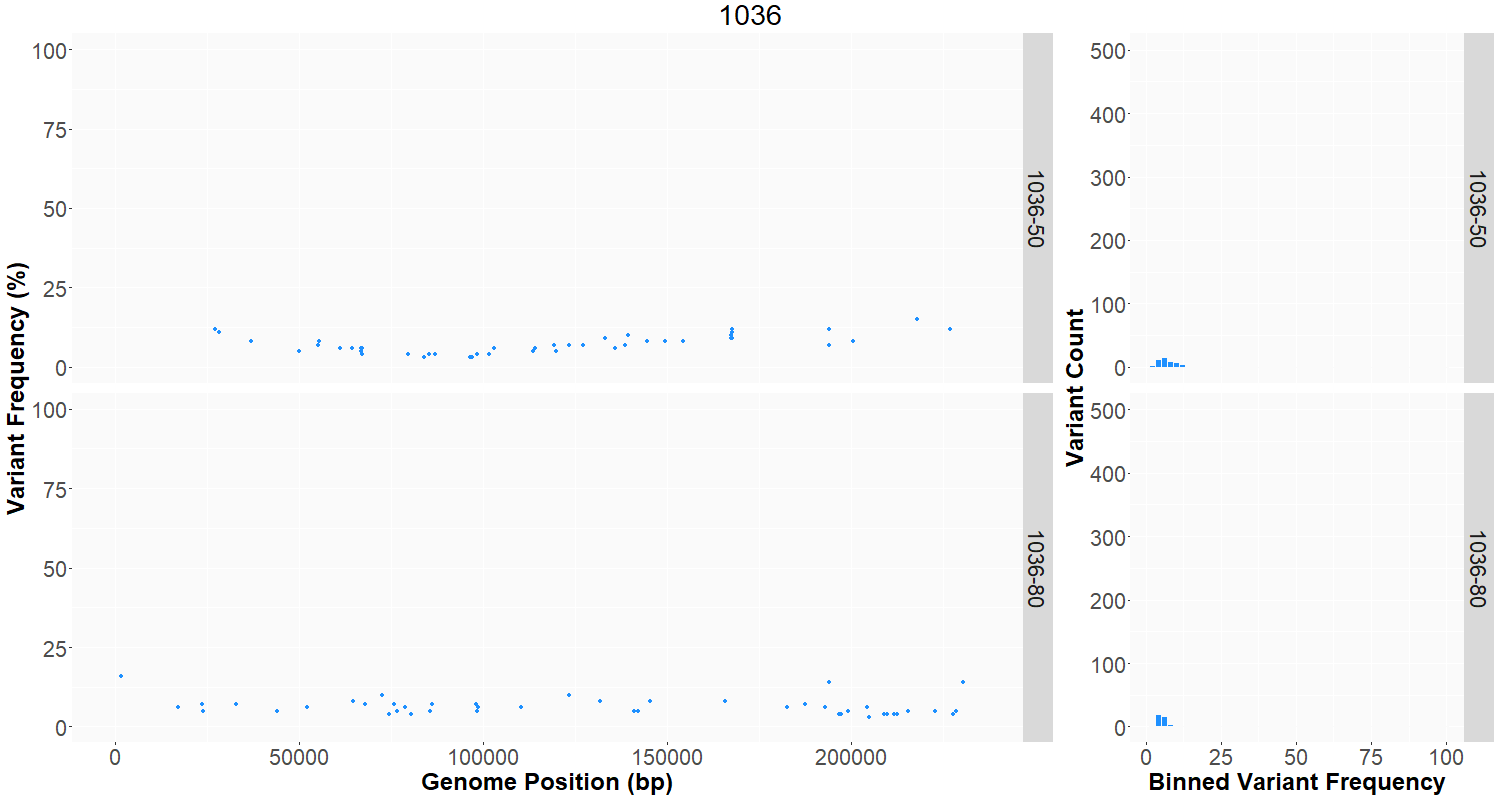

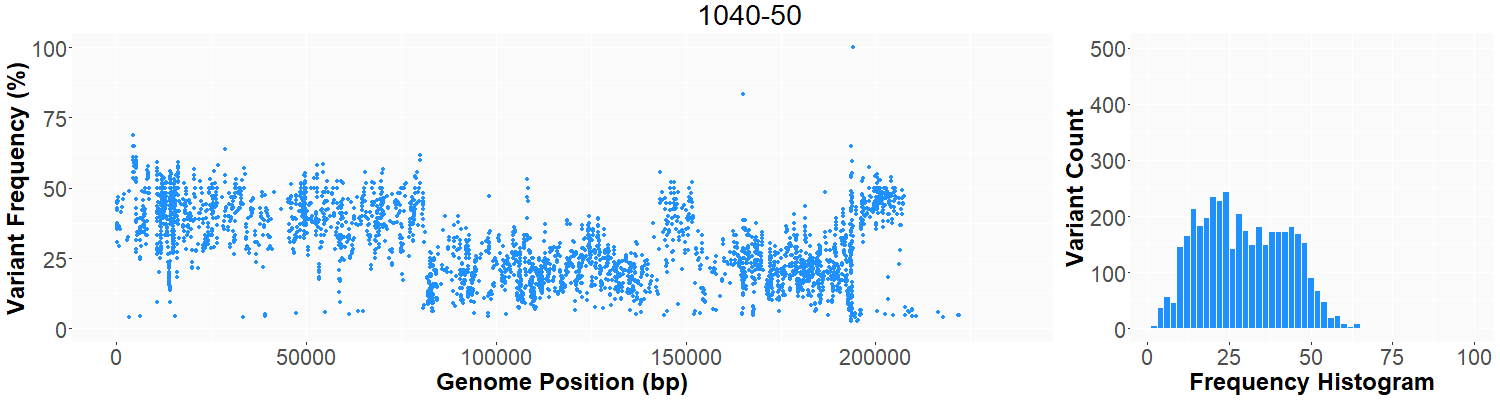

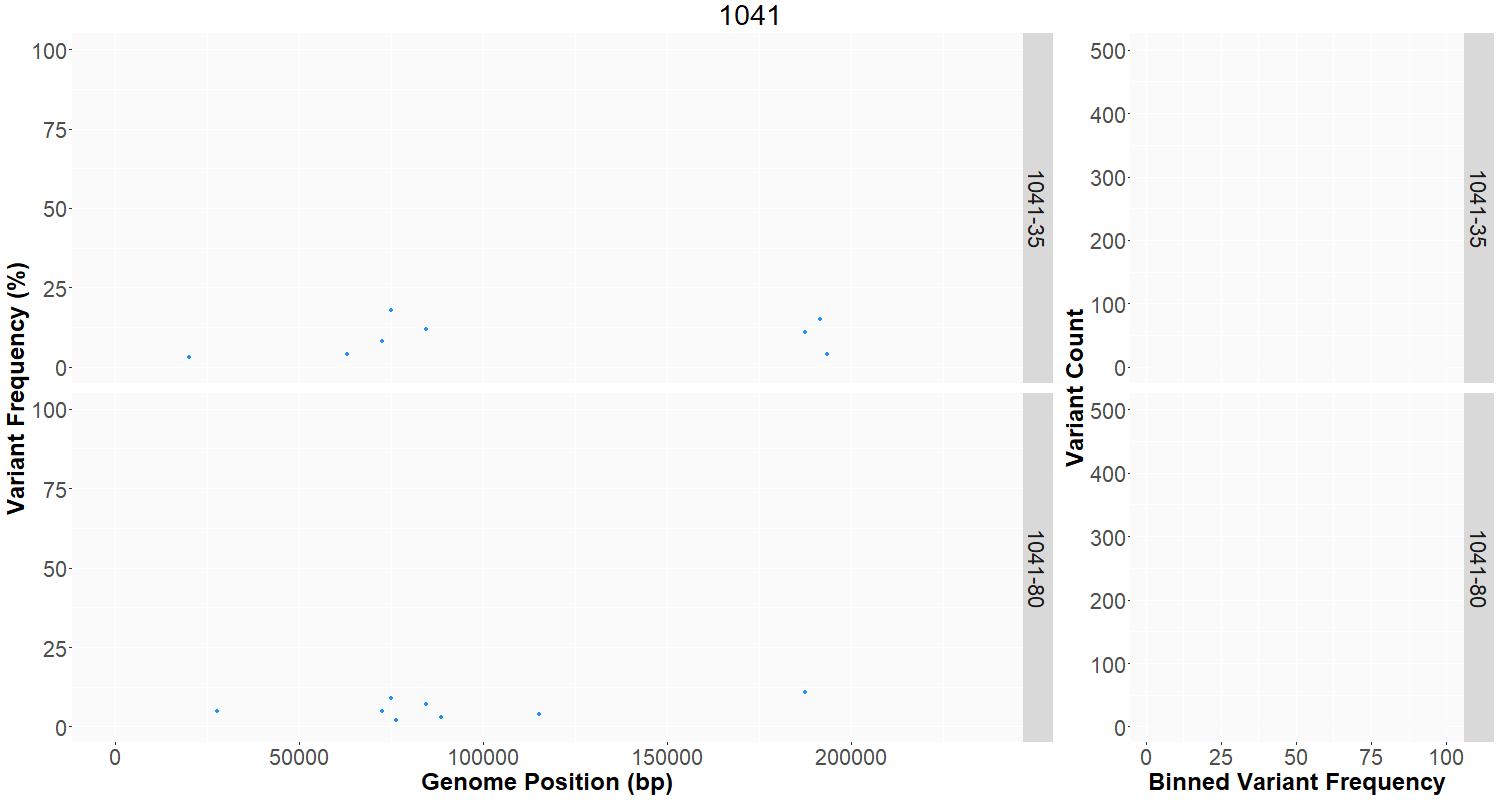

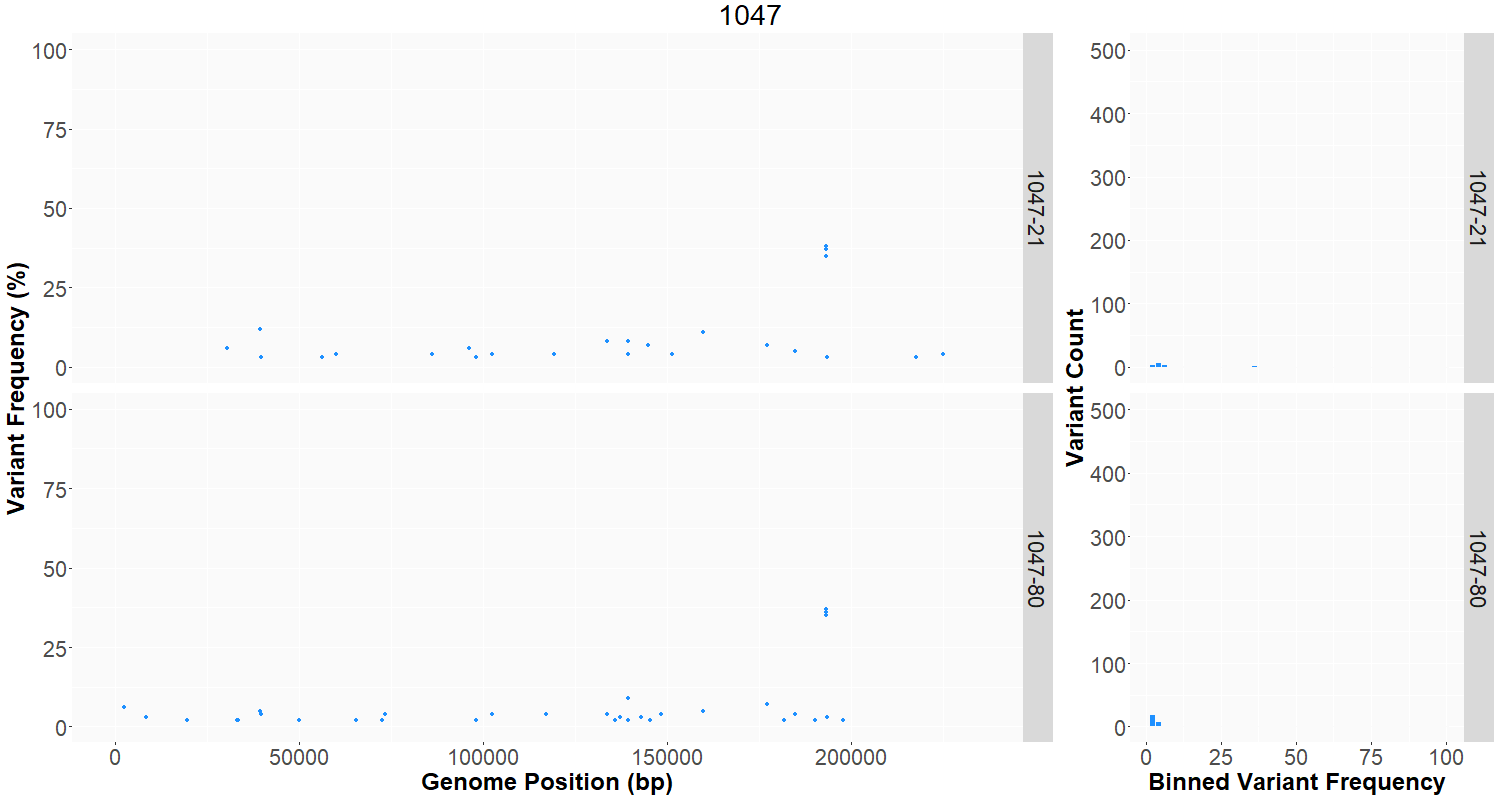

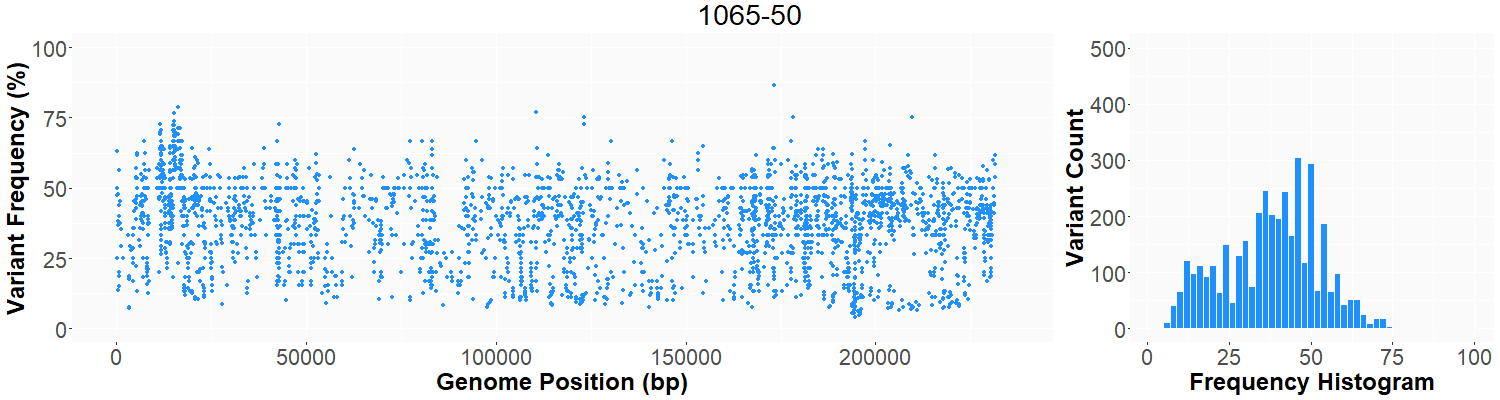

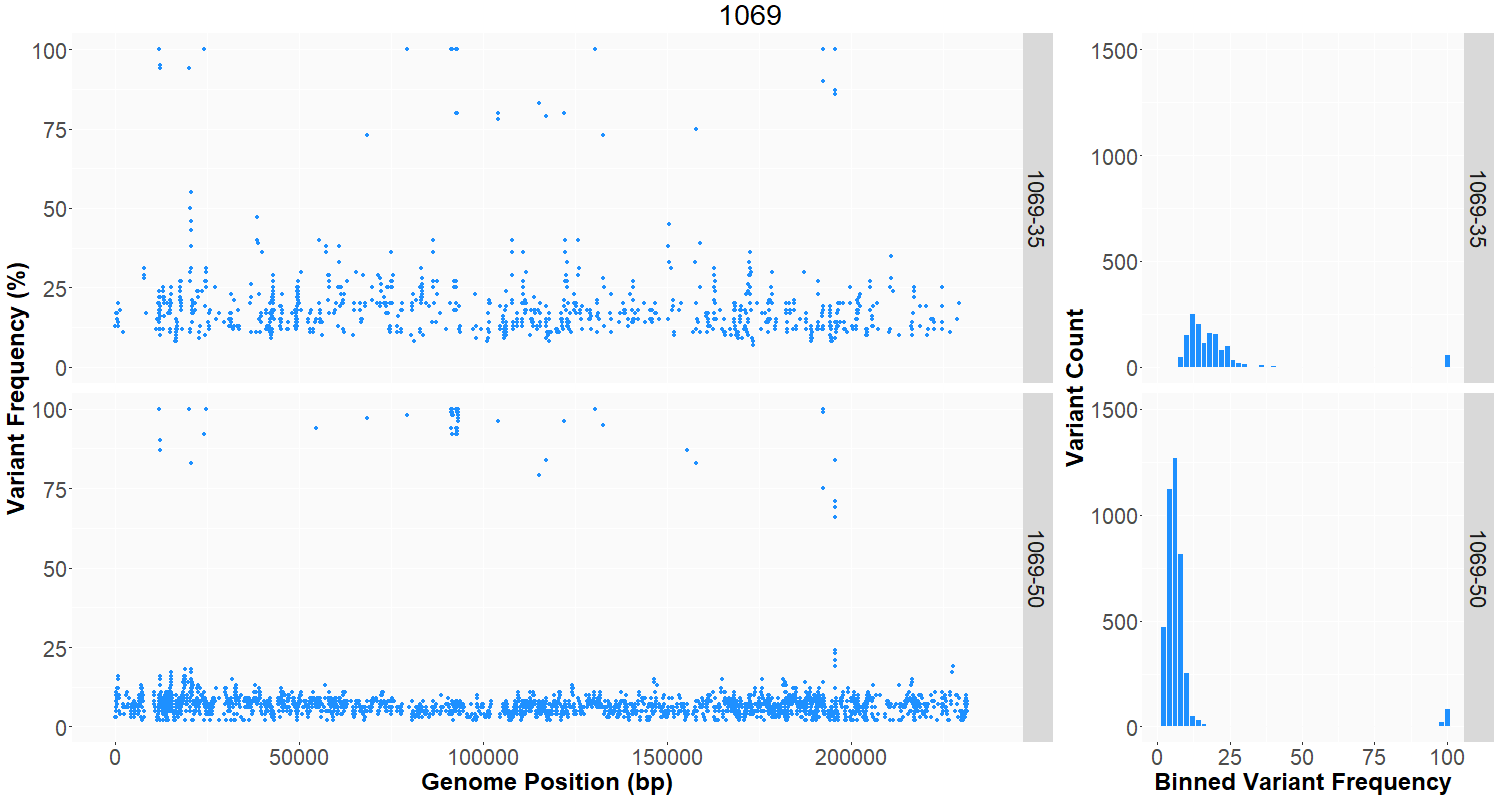

Supplement: Supplementary file 1 — Supplementary file1 (DOCX 292 KB) [file 430_2021_722_MOESM1_ESM.docx]
